# A method for measuring banana pseudo-stem phenotypic parameters based on handheld mobile LiDAR and IMU fusion

**DOI:** 10.3389/fpls.2024.1369501

**Published:** 2024-06-26

**Authors:** Zhou Yang, Qiming Jiang, Jieli Duan, Mohui Jin, Han Fu, Xing Xu

**Affiliations:** ^1^ Guangdong Laboratory for Lingnan Modern Agriculture, Guangzhou, China; ^2^ College of Engineering, South China Agricultural University, Guangzhou, China; ^3^ School of Mechanical Engineering, Guangdong Ocean University, Zhanjiang, China; ^4^ College of Electronic Engineering (College of Artificial Intelligence), South China Agricultural University, Guangzhou, China

**Keywords:** banana pseudo-stem diameter, banana pseudo-stem height, mobile LiDAR system, 3D point cloud, multi sensor fusion

## Abstract

Diameter and height are crucial morphological parameters of banana pseudo-stems, serving as indicators of the plant’s growth status. Currently, in densely cultivated banana plantations, there is a lack of applicable research methods for the scalable measurement of phenotypic parameters such as diameter and height of banana pseudo-stems. This paper introduces a handheld mobile LiDAR and Inertial Measurement Unit (IMU)-fused laser scanning system designed for measuring phenotypic parameters of banana pseudo-stems within banana orchards. To address the challenges posed by dense canopy cover in banana orchards, a distance-weighted feature extraction method is proposed. This method, coupled with Lidar-IMU integration, constructs a three-dimensional point cloud map of the banana plantation area. To overcome difficulties in segmenting individual banana plants in complex environments, a combined segmentation approach is proposed, involving Euclidean clustering, Kmeans clustering, and threshold segmentation. A sliding window recognition method is presented to determine the connection points between pseudo-stems and leaves, mitigating issues caused by crown closure and heavy leaf overlap. Experimental results in banana orchards demonstrate that, compared with manual measurements, the mean absolute errors and relative errors for banana pseudo-stem diameter and height are 0.2127 cm (4.06%) and 3.52 cm (1.91%), respectively. These findings indicate that the proposed method is suitable for scalable measurements of banana pseudo-stem diameter and height in complex, obscured environments, providing a rapid and accurate inter-orchard measurement approach for banana plantation managers.

## 1 Introduction

China ranks third globally in banana production and is a significant economic crop in southern regions, particularly in Hainan, Guangxi, and Guangdong (Zhang et al., 2008). However, banana cultivation practices in the country tend to be extensive, with a lack of modern production management techniques. This not only results in higher cultivation costs but also diminishes the competitiveness of banana yields and quality. Adult banana plants consist of components such as corms, pseudo-stems, leaves, and fruit clusters. The diameter and height of the banana plant pseudo-stem are crucial indicators for assessing plant health and predicting banana yields- (García et al., 2020; Magalhães et al., 2020). Accurately and rapidly measuring the pseudo-stem diameter and height is of paramount importance for evaluating banana growth and providing scientific guidance for field management.

Currently, cameras and LiDAR are the most commonly used devices for measuring crop phenotypic parameters (Wang et al., 2021). Wang et al. (Wang et al., 2023) employed a stereo vision camera to achieve rapid and non-destructive measurement of banana plant pseudo-stem diameter through binocular calibration, stereo rectification, and stereo matching. They utilized a cascade classifier for sample training. Song et al. (Song et al., 2019) proposed a 3D reconstruction algorithm using a depth camera. This algorithm employs a particle swarm optimization algorithm for pseudo-stem reconstruction, data fitting, and calculation of banana pseudo-stem diameter based on depth images obtained from an RGB-D camera. Peng et al. (Peng et al., 2022) utilized four different depth sensors, namely, Kinect V2, PMD CamBoard pico flexx, ZED stereo vision camera, and Velodyne 16-line LiDAR, to capture point clouds during the banana suckering stage. They extracted parameters such as plant height, stem diameter, and leaf area of banana suckers. Compared with manual measurements, Kinect V2 achieved optimal point cloud reconstruction and phenotypic parameter accuracy. Although cameras, compared with LiDAR, are cost-effective and provide more accurate measurement data, camera measurements in outdoor environments are influenced by environmental conditions such as lighting and weather. This may result in decreased image quality or the inability to obtain accurate information. Furthermore, cameras are unable to achieve large-scale measurement of banana pseudo-stem phenotypic parameters, posing challenges for the scale management of orchards.

Lidar technology offers advantages over camera devices in terms of measuring distance, accuracy, and environmental adaptability, making it widely applied in the field of crop phenotypic measurements (Peng et al., 2023). Miao et al. (Miao et al., 2022) proposed a method for measuring the diameter and height of banana pseudo-stems using ground-based terrestrial laser scanning (TLS). Similarly, Brack et al. (Brack et al., 2020) utilized TLS to acquire point cloud data in forests, measuring parameters such as tree diameter at breast height (DBH) and tree height. Shen et al (Shen et al., 2022), employing a robust deep learning framework, utilized TLS to obtain forest point cloud data and measured the DBH and tree height of wood in the forest. While TLS allows for large-scale and accurate measurement of some phenotypic parameters of trees, dense tree canopies or other obstructions around fruit tree trunks may hinder laser penetration, preventing the complete measurement of the trunk shape. Moreover, TLS devices often require the setup of scanning stations at different positions to obtain comprehensive trunk data (Gollob et al., 2019). This implies the need to identify suitable station positions and conduct multiple scans, increasing the complexity and workload of measurements. Given the complexity of ground-based lidar, some researchers have turned to mobile laser scanning (MLS for estimating tree morphological parameters). Su et al. (Su et al., 2020) developed a backpack LiDAR system for estimating phenotypic parameters such as tree diameter and height in the forest. The results showed that the backpack lidar system could accurately estimate tree height, comparable with TLS, without the need for multiple receiver stations, thereby improving efficiency. Bienert et al. (Bienert et al., 2021) used a mobile lidar platform to reconstruct three-dimensional landscapes in large forest areas and successfully extracted individual trees from point clouds. Čerňava et al. (Čerňava et al., 2019) fused GNSS data with a Riegl VMX-250 lidar scanner to build globally consistent point cloud data and estimate DBH. Yadav et al. (Yadav and Lohani, 2020) used the StreetMapper 360 mobile lidar scanning system, fused with GNSS data, to reconstruct urban road scenes and separate trees and their trunks. While MLS systems can provide real-time point cloud data of the environment, they typically require a mobile platform and GNSS data for assistance. In environments where it is difficult for mobile platforms to access and GNSS coverage is low, such as in dense canopies, MLS systems may fail to provide globally consistent point cloud data (Fan et al., 2018; Xu et al., 2019; Wu et al., 2020), thus hindering accurate construction of point cloud maps in the region.

In response to this situation, in recent years, laser SLAM (Simultaneous Localization and Mapping) technology has been widely applied to the measurement of crop phenotypic parameters, enabling simultaneous localization and mapping in real time within unfamiliar environments (Bailey and Durrant-Whyte, 2006; Durrant-Whyte and Bailey, 2006). SLAM is a critical technique that fuses perception and localization, utilizing sensors such as cameras and LiDAR to gather environmental information. Combined with motion estimation algorithms, SLAM generates maps and estimates the platform^′^s position simultaneously. Zhou et al. (Zhou et al., 2019) employed a VLP-16 LiDAR with the LiDAR Odometry and Mapping (LOAM) algorithm to generate a three-dimensional point cloud map of forest areas, fitting tree diameters at breast height (DBH) using the RANSAC algorithm. The mean absolute error of tree radius was 0.43 cm, with an overall relative error of 2.27%, meeting forestry mapping requirements. Pierzchała et al. (Pierzchała et al., 2018) utilized a graph-SLAM-based simultaneous localization and mapping algorithm to generate local maps of forests, evaluating the accuracy of fitting diameter at breast height (DBH). The aforementioned approaches involve the use of single sensors or loosely coupled methods for laser mapping, which can lead to issues such as trajectory drift and mapping failures in complex outdoor environments. In recent years, Lidar-IMU tightly coupled methods, such as the notable LIO-Mapping (Ye et al., 2019) and LIO-SAM (Shan et al., 2020), have significantly improved mapping accuracy and stability, providing reliable methods for measuring crop phenotypic parameters. This study adopts the robust LIO-SAM mapping and localization algorithm for three-dimensional reconstruction of environmental maps.

Utilizing a handheld mobile LiDAR system in conjunction with synchronized mapping and localization methods presents a versatile and portable solution for mobile laser scanning. We adopted the LIO-SAM laser SLAM algorithm as the foundational framework for our three-dimensional map reconstruction algorithm; this approach allows for easy deployment in various environments, offering real-time acquisition of high-precision three-dimensional point cloud data. In response to the need for measuring morphological parameters of banana pseudo-stems, this study introduces a Lidar-IMU fusion SLAM data collection platform. Initially, the mobile LiDAR and IMU sensors are integrated to construct a three-dimensional orchard map for banana plantations. Addressing challenges faced by LIO-SAM in outdoor orchard environments, where irregular shapes of branches and leaves, as well as measurement noise, can lead to inaccurate, incomplete, and uneven feature extraction, a distance-weighted feature extraction method is proposed. This method enhances the accuracy and robustness of mapping the banana plantation environment by extracting edge and plane features effectively. Subsequently, a segmentation process is applied using a combined approach of filtering, Euclidean clustering, and K-means clustering to isolate the point clouds of individual banana plants. Finally, a sliding window method is introduced to extract essential morphological parameters such as the diameter and height of the banana pseudo-stems. The main contributions of this study include (1) the development of a handheld mobile LiDAR inertial navigation fusion collection platform applied to the measurement of banana pseudo-stem phenotypic parameters; (2) the introduction of a distance-weighted principal component feature extraction method; and (3) the proposal of an automated measurement method for calculating the diameter and height of banana pseudo-stems.

## 2 Methodology

**Figure 1:**
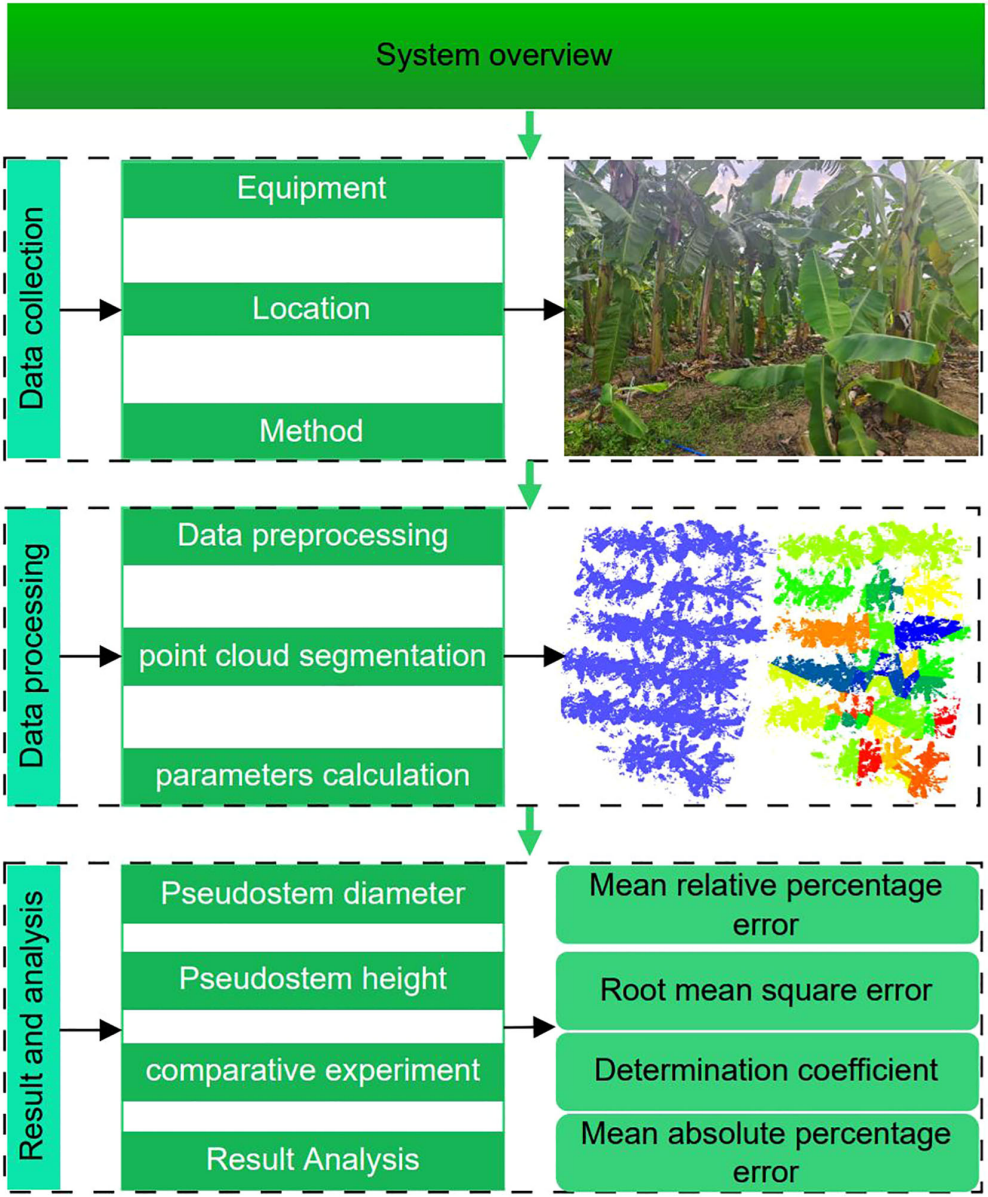
System overview.

The entire banana pseudo-stem diameter and height measurement system primarily consists of three parts, data acquisition, point cloud data processing, and phenotypic parameter measurement, as illustrated in **Figure 1**. Taking banana plants as the research subject, we integrate data from mobile LiDAR and inertial measurement unit (IMU) to perform three-dimensional reconstruction of the environmental map. The acquired environmental point cloud undergoes preprocessing, including ground removal and cluster segmentation, to separate each banana plant and measure its pseudo-stem diameter, height, and other phenotypic parameters. The measured phenotypic parameters are compared with manually obtained true values to assess the measurement accuracy of the algorithm.

### 2.1 Data acquisition

#### 2.1.1 Collection equipment and locations

**Figure 2:**
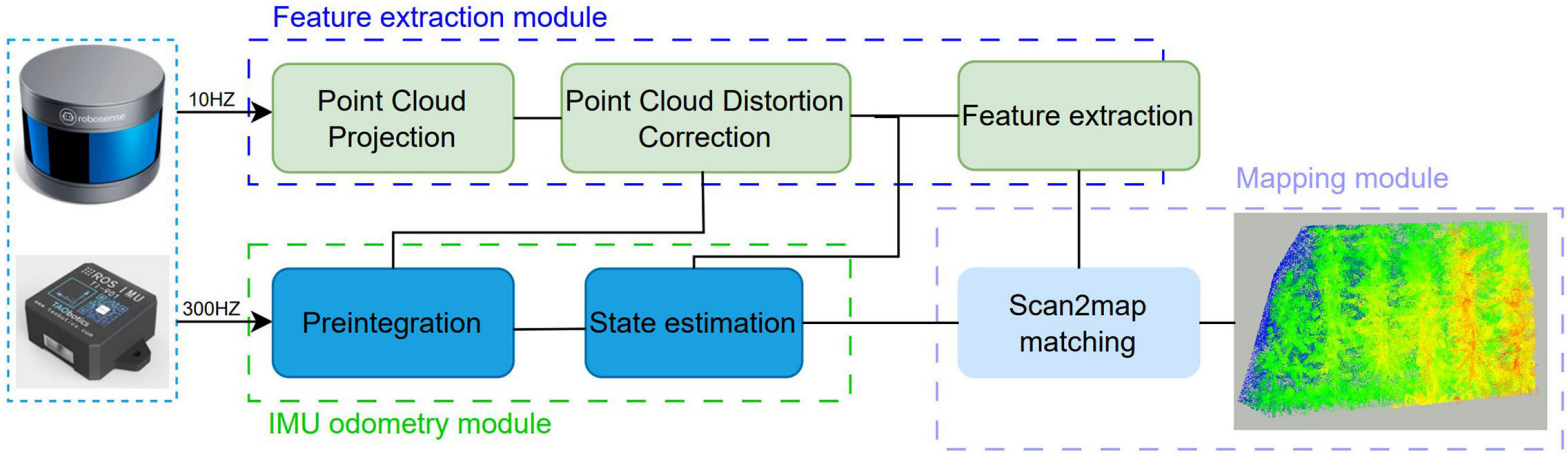
Data collection framework.

The data acquisition system is illustrated in **Figure 2**. The handheld 3D scanning device used is the RoboSense RS-16 LiDAR, coupled with the HFI-A9 nine-axis IMU by HandsFree Robotics. The laptop employed is the Lenovo Legion Y7000(i5–6300HQ), featuring a quad-core processor, a quad-thread central processing unit (CPU), 8 GB of DDR4 RAM, and a 512-GB SSD hard drive. The experimental data were collected at the banana orchard of the Teaching and Research Base of South China Agricultural University, covering an area of approximately 30 × 30 m and including 43 banana trees.

### 2.2 Data acquisition method

#### 2.2.1 IMU odometry module

1) IMU preintegration: The measured values of angular velocity and acceleration output by the IMU sensor are defined as follows in Equations 1 and 2:


(1) $$\tilde{\omega }=\omega^{b}+b^{g}+n^{g}\;$$

(2) $${\tilde{a}}^{b}=q_{bw}\left(a^{\omega }+g^{\omega }\right)+b^{a}+n^{a}$$

The variable b represents the random walk bias; n denotes random white noise; *q_bw_* represents the rotation matrix from the world coordinate system to the IMU body coordinate system, and g is the gravity vector in the world coordinate system. We collect measurements within the time interval[i, j]. Based on the original measurement data collected within the time interval[i, j], we can infer the robot’s motion using Equations 3, 4 and 5.


(3) $$R_{j}=R_{i}\cdot \prod_{k=i}^{j-1}Exp\left(\left(\tilde{\omega }-b_{k}^{g}-\eta_{k}^{gd}\right)\text{Δ}t\right)$$

(4) $$v_{j}=v_{i}+g\text{Δ}t_{ij}+\sum_{k=i}^{j-1}R_{k}\cdot \left(\tilde{f_{k}}-b_{k}^{a}-\eta_{k}^{ad}\right)\cdot \text{Δ}t$$

(5) $$P_{j}=P_{i}+\sum_{k=i}^{j-1}\left[V_{k}\cdot \text{Δ}t+\frac{1}{2}g\cdot \text{Δ}t^{2}+\frac{1}{2}R_{k}\cdot \left(\tilde{f_{k}}-b_{k}^{a}-\eta_{k}^{ad}\right)\text{Δ}t^{2}\right]$$

In the above, $R_{j}\;、V_{j}$ , and *P_j_* represent the azimuth, velocity, and position at time *j*, respectively, estimated based on the state at time *i*. We employ the method outlined in the literature Forster et al. (2016) to compute the relative motion between two instants, denoted as Σ*R*, Σ*V*, and Σ*P*, and integrate these values into the state estimation.

2) State estimation: The transformation of the inertial measurement unit (IMU) states at time i and time j is defined as $x_{b_{i}}^{b_{j}}$ , and the residual during the transformation is represented by $\delta_{x}$ as shown in Equations 6 and 7.


(6) $$x_{b_{i}}^{b_{j}}=\left[p_{j}^{i},v_{j}^{i},q_{j}^{i},b_{a},b_{g},g^{k}\right]$$

(7) $$\delta_{x}=\left[\delta_{p},\delta_{v},\delta_{\theta },\delta_{b},\delta_{a},\delta_{g}\right]$$

In the equations, $x_{bi}$ represents the state of the IMU in the body coordinate system at time i; $p_{j}^{i}$ and $q_{j}^{i}$ denote the translation and rotation of the IMU from time i to j; $b_{a}$ and $b_{g}$ represent accelerometer and gyroscope biases; $g^{k}$ represents the gravity vector. The continuous-time motion and noise transfer for the IMU are described in Equations 8 and 9, respectively. This paper employs the iterative Kalman filtering method proposed by Zheng et al. (2018) to propagate errors and obtain range information. Equations 10–12 represent its error propagation and update model.


(8) $$x_{j}=F_{j-1}x_{j-1}+G_{j-1}\mu_{j-1}$$

(9) $$\delta_{j}=F_{j-1}\delta j-1+G_{j-1}n$$

In the equations, $x_{bi}$ represents the state of the Inertial Measurement Unit (IMU) in the body coordinate system at time i; $p_{j}^{i}$ and $q_{j}^{i}$ denote the translation and rotation of the IMU from time i to j; $b_{a}$ and $b_{g}$ represent accelerometer and gyroscope biases; and $g^{k}$ represents the gravity vector. The continuous-time motion and noise transfer for the IMU are described in Equations 8 and 9. This paper employs the iterative Kalman filtering method proposed by Zheng et al. (Zheng et al., 2018) to propagate errors and obtain range information.


(10) $$K_{j}=P_{j|j-1}H_{j}^{T}{\left(H_{j}P_{j|j-1}H_{j}^{T}+J_{j}M_{i}J_{j}^{T}\right)}^{-1}$$

(11) $$\delta_{x_{j}}=\delta_{x_{j-1}}+K_{i,j}\left(H_{i,j}\delta_{x_{j-1}}-f\left(x_{b_{i}}^{b_{j}}⊕\delta_{x}\right)\right)$$

(12) $$P_{j|j-1}=F_{j-1}P_{j-1|j-1}F_{j-1}^{T}+Q_{j-1}$$

After completing the iteration, obtain the optimal estimate of the current state, and update the covariance matrix $P_{j|j}$ using Equation 13. Then, initiate the next iteration.


(13) $$P_{j|j}=\left(I-K_{j}H_{j}P_{j|i}\right)$$

#### 2.2.2 Feature extraction module

1) Point cloud projection and distortion correction: Taking the example of the laser radar in this paper, operating at a frequency of 10 Hz with a scanning period of approximately 0.1 s. Within one scanning period, points in the point cloud are not acquired at the same moment due to the motion of the laser radar with the carrier. In other words, the coordinate systems of different laser points in the same frame are inconsistent. Therefore, distortion correction is necessary for the point cloud. For points within the time span [t, t+0.1], the initial pose of the robot has been obtained in Section 2.2.1. This pose is considered as the initial pose at time t. However, the IMU outputs data at a frequency of 300 Hz for angular velocity and acceleration. To better fuse data of two different frequencies, we employ a linear interpolation method as shown in Equation 14 to interpolate the 10-Hz lidar data onto the time steps of the 300-Hz IMU pose data, facilitating alignment with the pose data obtained from IMU preintegration.


(14) $$P_{d}\overset{\text{Δ}}{=}ℜ\left(slerp\left(q{,}_{t_{k}^{"}}^{t_{k}}\overset{⌣}{q},s\right)\right)P_{d}+s_{t_{k}^{\prime }}^{t_{k}}\overset{⌣}{p}$$

where $s=\frac{t_{s}}{t_{k}^{,-}t_{k}}$ ; slerp(q1,q2,s) is the spherical linear interpolation operation on quaternions (Dam et al., 1998).

2) Distance-weighted feature extraction method: When a frame of LiDAR point cloud data is received, we project the point cloud obtained from LiDAR into a distance image (image resolution of 16 × 1,800) for the extraction of point cloud features. In contrast to the method proposed by Shan et al. (2020), which relies on roughness for feature extraction, we adopt a different approach. For a given frame of LiDAR point cloud P, where each point $p_{i}=\;{\left[x_{i},y_{i}z_{i}\right]}^{T}$ , we identify m points in its left and right neighborhoods, denoted as $\left[p_{i-m},\ldots .,p_{i-1}\right]$ and $\left[p_{i}+1,\ldots .,p_{i}+m\right]$ , respectively. We then compute the centroid coordinates $P_{mean}$ of the frame’s point cloud using Equation 15 and the covariance matrix C for each point as shown in Equation 16.


(15) $$P_{mean}=\frac{1}{2m+1}\sum_{K=i-m}^{i+m}P_{K}$$

(16) $$C=\frac{1}{2m}\sum_{i=1}^{m}\left(W_{jmean}*{\left(P_{j}-P_{mean}\right)}^{T}\left(P_{j}-P_{mean}\right)\right)$$

where $W_{jmean}$ represents the weight, determined by calculating the distance from the current point to the new centroid coordinates. M is a 3 × 3 matrix, and performing the eigenvalue decomposition on the covariance matrix C yields three eigenvalues $\lambda_{1},\lambda_{2}$ , and $\lambda_{3}\left(\lambda_{1}>\;\lambda_{2}>\;\lambda_{3}\right)$ . Define $F_{i}^{e}$ and $F_{i}^{p}$ as the line feature points and plane points at time i, respectively. The features of the point cloud are extracted using Equation 17.


(17) $$\lambda =\lambda_{1}+\lambda_{2}$$

If the *λ* value is less than the threshold $E_{t}$ , then the point is classified as a linear feature point. If the *λ* value is greater than the threshold $P_{t}$ , then it is classified as a planar point. For all the features extracted at time i, they form a LiDAR frame $F_{i}$ , where $F_{i}$ = { $F_{i}^{e}$ , $F_{i}^{p}$ }.

#### 2.2.3 Mapping module

To improve the accuracy of the composition, we employ a scan matching method to find the optimal pose of the current laser frame to the already constructed map. We scan match the newly acquired laser frame $F_{i}^{e}$ , $F_{i}^{p}$ with $M_{i}$ . Various scan matching methods can be utilized (Besl and McKay, 1992; Segal et al., 2009; Zhang and Singh, 2017), and we choose the method proposed in Zhang and Singh (2017) due to its computational efficiency and robustness in challenging environments. Using the pose transformation matrix *T_i_* estimated in Section 2.11, we transform $F_{i}^{e}$ , $F_{i}^{p}$ from the local coordinate system to the world coordinate system, obtaining $F_{i}^{we}$ , $F_{i}^{wp}$ . We then find corresponding matches in $M_{i}^{e}$ and $M_{i}^{p}$ , and upon successful matching, insert them into the map. The distance between features and their corresponding edges or planar patches can be calculated using the Equations 18 and 19.


(18) $$d_{ek}=\frac{\left|\left(p_{i+1,k}^{e}-p_{i,u}^{e}\right)\times \left(p_{i+1,k}^{e}-p_{i,v}^{e}\right)\right|}{\left|p_{i,u}^{e}-p_{i,v}^{e}\right|}$$

(19) $$d_{pk}=\frac{\left|\begin{array}{c} p_{i+1,k}^{p}-p_{i,u}^{p}\\ \left(p_{i,u}^{p}-p_{i,v}^{p}\right)\times \left(p_{i,u}^{p}-p_{i,w}^{p}\right) \end{array}\right|}{\left|\left(p_{i,u}^{p}-p_{i,v}^{p}\right)\times \left(p_{i,u}^{p}-p_{i,w}^{p}\right)\right|}$$

Here, k, u, v, and w are the feature indices corresponding to the feature set. For the line feature set $F_{i+1}^{we}$ with $p_{i+1,k}^{e}$ , we calculate its straight-line distance $d_{ek}$ to the two nearest features $p_{i,u}^{e},p_{i,v}^{e}$ in the local map line feature set $M_{i}^{e}$ . For the plane feature set $F_{i+1}^{wp}$ with $p_{i+1,k}^{p}$ , we calculate its plane distance $d_{pk}$ to the nearest features $p_{i,u}^{p},p_{i,v}^{p}$ , and $p_{i,w}^{p}$ in the local map plane feature set $M_{i}^{p}$ . By minimizing the distances $d_{ek}$ and $d_{pk}$ , we can obtain the optimal position of the new feature point in the map. This establishes a least squares problem as formulated in Equation 20, and the Gauss–Newton method is used to solve this nonlinear least squares equation.


(20) $$\begin{array}{c} \text{min }\\ T_{i+1} \end{array}=\left\{\sum_{p_{i+1,k}^{e}\in F\leq_{i+1}^{we}}d_{ek}+\sum_{p_{i+1,k}^{p}\in F_{i+1}^{wp}}d_{pk}\right\}$$

### 2.3 Banana Grove point cloud data processing

**Figure 3:**
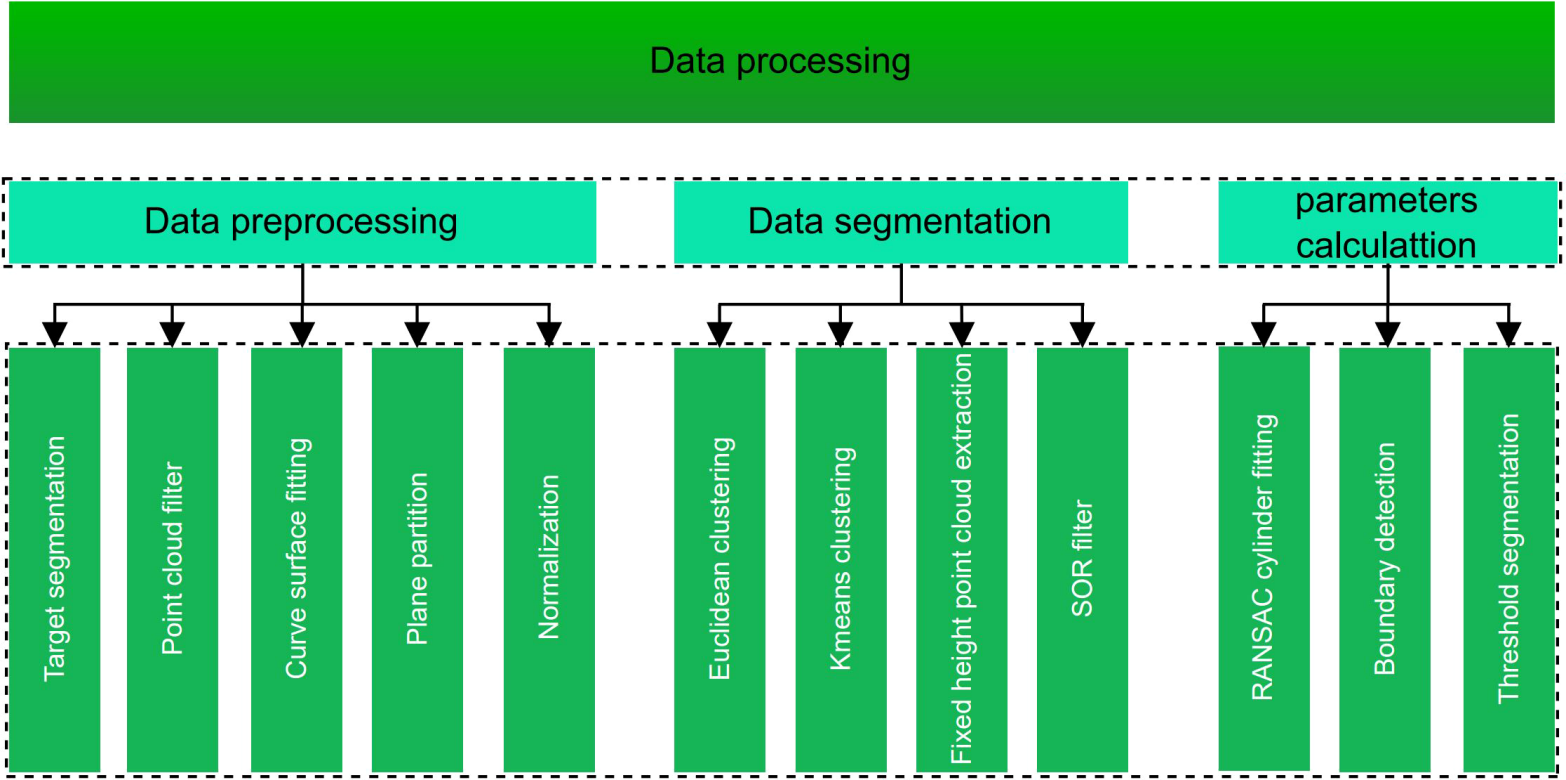
Point cloud data processing framework.

Utilizing CloudCompare software as a three-dimensional point cloud data processing tool, we perform basic preprocessing on the point cloud obtained after mapping. Using Visual Studio 2015 as the platform, with the installation of Point Cloud Library 1.8.0 (PCL 1.8.0) and CMake 4.1.2, we implement automated and rapid measurement of banana pseudo-stem diameter, height, and other phenotypic parameters through C++ programming. The three-dimensional point cloud data processing consists of three main parts: data preprocessing, point cloud segmentation, and pseudo-stem phenotypic parameter measurement. The overall framework of point cloud data processing is illustrated in **Figure 3**.

#### 2.3.1 Point cloud preprocessing

The data preprocessing process includes several steps: target area segmentation, point cloud filtering, surface fitting, elevation normalization, and plane segmentation. The point cloud data collected in the experiment are read, and the target area is segmented using CloudCompare software. The generated point cloud map typically contains some noise points. To address this, statistical filtering in CloudCompare software is employed to remove noise points, preventing its impact on the measurement parameters. Fruit trees and the ground are not separated. This can affect subsequent clustering. Considering the unevenness of the experimental environment, especially the rough terrain, this study initially applies the Cloth Simulation Filter (CSF) algorithm (Zhang et al., 2016) to fit the mesh of the ground point cloud. The distance from each point in the original point cloud to the ground mesh is calculated, and elevation normalization is performed in the Z-axis direction, establishing a uniform lowest point for subsequent tree trunk height calculations. After normalization, the ground is fitted into a plane. The Random Sample Consensus (RANSAC) plane segmentation algorithm (Schnabel et al., 2007) is then employed to segment the ground point cloud data.

#### 2.3.2 Banana tree point cloud segmentation

To facilitate the rapid calculation of banana pseudo-stem diameter and height, an automated method is developed based on C++. This method includes operations such as extracting high points from the point cloud, performing Euclidean clustering, K-means clustering, and statistical filtering, as outlined in **Algorithm 1**. This algorithm is designed for the segmentation of banana tree point clouds.

**Algorithm 1:** Segmentation method of point cloud.

**Input:** *P_ori_ *: A pre-processed point cloud; *T_e_ *: Euclidean clustering distance threshold; T: Width threshold; **Output:** *P_s_ *: A set of single banana point cloud; 1 Initialization: *P_s_ * ← *ϕ*; n=1; 2 Extract point cloud *P_f_ * of plane [1.0, 1.2]m from *P_ori_ *; 3 Statistically filtered *P_f_ *; 4 Euclidean clustering *P_f_ * generated *P_fe_ *; 5 **for** *i* ← 1 **to** *size(P _fe)* **do** 6 *I*nitialization K← 0; 7 *K = (x_max_ *-*x_min_)+(y_max_-y_min_)*; 8 **if *K>T* then** 9 Statistically filtered the i *P_fe_ *; 10 Save the i clustered point cloud *P_fe_ *; 11 Calculate centroid using Centroid calculation formula; 12 Save (K, centroid) as an array; 13 **end** 14 **end** 15 Input (K,centroid) and *P_fe_ *; 16 Kmeans clustering; 17 Save cluster point cloud *P_fek_ *;

After removing the ground point cloud, the point cloud of banana plants in the experimental area is not separated individually and needs to undergo clustering segmentation. Common traditional point cloud clustering segmentation algorithms include region growing segmentation (Nurunnabi et al., 2012), minimum cut-based segmentation (Golovinskiy and Funkhouser, 2009), normal difference-based segmentation (Ioannou et al., 2012), super-voxel-based segmentation (Papon et al., 2013), progressive morphological filtering segmentation (Zhang et al., 2003), Euclidean clustering extraction algorithm (Sun and Salvaggio, 2013), density-based spatial clustering of applications of noise (DBSCAN), and K-means clustering algorithm (MacQueen, 1967). Considering the spatial distribution characteristics of banana plants in the plantation and the clustering effects, parameter adjustments, and time consumption of other algorithms, this study ultimately employed a combination of the euclidean clustering and threshold segmentation and the K-means clustering algorithm method to identify banana pseudo-stem point clouds. However, the K-means algorithm is significantly influenced by the number of categories (K) and the starting points of clustering, so these two parameters should be determined first.

### 2.4 Calculation of banana pseudo-stem phenotypic parameters

#### 2.4.1 Calculation of banana pseudo-stem diameter

The measurement of the diameter of a banana pseudo-stem involves two parts: the extraction of a single pseudo-stem’s point cloud at a fixed height and the calculation of the pseudo-stem’s diameter. In banana pseudo-stem diameter measurement, positions 1 m above the ground are typically extracted for measurement (Miao et al., 2022). Considering the growth conditions of banana plants in the experimental area and the influence of ground weeds and leaves, this study extracts point clouds in the range of 1.0 m–1.2 m above the ground for calculating the pseudo-stem diameter. In the previous section, the segmentation extraction and filtering of a single banana pseudo-stem point cloud were completed, as shown in **Algorithm 1**. For the measurement of banana pseudo-stem diameter, different methods exhibit varying levels of robustness in their measurement results. Hence, we compared four different circle fitting methods for estimating the size of banana pseudo-stems: Least Squares Circle Fitting (Chernov and Lesort, 2005), Hough Transform Circle Fitting (Rabbani and Van Den Heuvel, 2005), Robust Least Trimmed Squares Method (Nurunnabi et al., 2017), and RANSAC Circle Fitting (Kuželka et al., 2020). Least Squares Circle Fitting is a classical approach that minimizes the sum of squared distances between data points and the fitted circle. While computationally simple, its accuracy may be compromised when dealing with noisy or outlier-prone data. Originally designed for detecting circular shapes in images, Hough Transform Circle Fitting has been adapted for two-dimensional point cloud circle fitting. It detects and fits circular objects in point cloud data, but its performance can be sensitive to preprocessing and parameter selection, potentially leading to computational overhead and unstable results. Robust Least Trimmed Squares Method is a variant of Least Squares Circle Fitting that enhances robustness by removing outliers with significant influence on the fitting result. This method is suitable for scenarios with noise or outliers, providing a more robust circle fitting. RASNSAC Circle Fitting employs the Random Sample Consensus (RANSAC) algorithm to fit circles. By iteratively selecting random subsets of data points for fitting and selecting the best fitting result, it demonstrates strong robustness against noise and outliers.

#### 2.4.2 Calculation of banana pseudo-stem height

**Figure 4:**
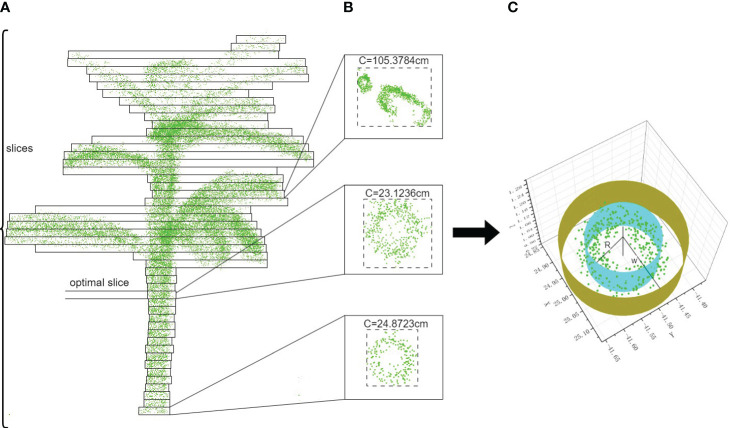
Stem-leaf junction recognition process. **(A)** The process of sliding window slicing. **(B)** Calculation of the perimeter for different slicing windows. **(C)** Threshold segmentation diagram.

The measurement of the pseudo-stem height of bananas involves three main components: threshold segmentation, boundary point recognition, and height measurement. The key to pseudo-stem height measurement lies in determining the intersection points between the pseudo-stem and the leaf, as well as between the pseudo-stem and the ground. The height difference along the axial direction between these two positions is considered as the height. The pseudocode for the entire pseudo-stem height measurement is outlined in **Algorithm 2**. In **Algorithm 2**, to accurately identify the boundary points between the banana pseudo-stem and the leaves, a threshold segmentation method is initially employed to obtain the point cloud near the banana pseudo-stem. Specifically, by calculating the distance of each point in the current banana plant to the directional vector, if this distance is less than the threshold W, the point is retained; otherwise, it is removed. The segmentation process is illustrated in **Figure 4C**. Subsequently, a continuity-based sliding window approach is proposed to identify the boundary points between the pseudo-stem and the leaves. After threshold segmentation, the point cloud is horizontally sliced from bottom to top, as shown in **Figure 4A**. The thickness of the slice should not be too large.

**Algorithm 2:** Measurement of banana pseudostem height.

**Input:** $P_{fek}$ : point cloud after kmeans clustering; R: pseudostem radius; V: pseudostem axis vector; d: window distance; W: threshold; $d_{w}$ : threshold; **Output:** banana pseudostem height H; 1 Initialization: H ← ϕ ; 2 **for** i←1 **to** *size*( $P_{fe}$ ) **do** 3 Set W: 0.15m; set d: 0.02m; set $d_{w}$ : 0.1m; 4 Threshold segmentation of $P_{f}ek$ ; 5 Slice the point cloud $P_{fek}$ into different layers $P_{L}$ ; 6 Set the continuous flag as 0; 7 **for** i←1 **to** *size* ( $P_{L}$ ) **do** 8 Calculate distance a= $X_{max}$ - $X_{min}$ ; 9 Calculate distance b= $Y_{max}$ - $Y_{min}$ ; 10 **if** a+b *¿* 4 *R + $d_{w}$ **then** 11 continuous flag+; 12 **if** continuous flag > *10* **then** 13 $Z_{max}$ = $star_{position}$ + $d_{w}*\left(window-8\right)$ ; 14 **else** 15 Continuous flag ← 0; 16 **end** 17 **end** 18 **end** 19 **end**

Through experimentation, it has been validated that setting the distance d of the sliding window, i.e., the slice thickness, to 0.02 m is optimal. As determined from the earlier calculation of pseudo-stem diameter, slicing the pseudo-stem horizontally at a height of 0.5 m above the ground is not affected by weeds, leaves, etc. Therefore, starting from a height of 0.5 m above the ground, all point clouds of the banana plant are segmented into different windows. The lengths a and b of each window in the X and Y directions are calculated. By assessing the relationship between the window’s perimeter C and its diameter, the occurrence of a perimeter mutation in the window can be determined, as shown in **Figure 4B**. On the XY plane, the perimeter of the boundary point between the stem and leaf is greater than that of the pseudo-stem edge point cloud. Hence, by calculating the perimeters of point clouds in the XY plane for different windows, positions with perimeter mutations can be identified, indicating potential boundary points between the pseudo-stem and the leaves. When the perimeter mutations persist for 10 consecutive windows, it can be concluded that a boundary point has been found, with the window of the first occurrence in the current continuous sequence being identified as the boundary point window. Since the lowest point in the Z direction has been standardized for all plants, the point cloud at the intersection between the banana plant and the ground represents the minimum value in the Z direction for the pseudo-stem, and each plant’s lowest point is the same, located at the lowest point of the pseudo-stem point cloud.

## 3 Result and discussion

### 3.1 Feature extraction module comparative experiment

**Figure 5:**
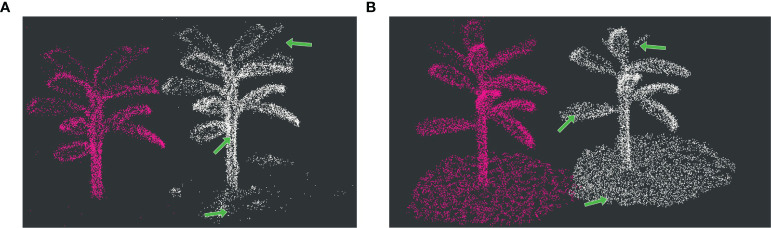
Feature extraction comparative diagram: **(A)** Depicts a line feature comparison; **(B)** illustrates a planar feature extraction comparison. In both cases, the white point cloud represents the reconstructed point cloud using the original method, whereas the pink point cloud represents the point cloud reconstructed using the method proposed in this paper.

To evaluate the effectiveness of the distance-weighted feature extraction method, we utilized the data storage functionality in ROS to save the reconstructed maps. For better visualization, we opted to record the map reconstructed from a single banana tree instead of using the entire experimental banana plantation map for visualization. The comparative experiments primarily focused on assessing the differences in the performance of the reconstructed point clouds at edge and planar points. The point cloud maps constructed using the original method and the improved method are depicted in the **Figure 5**. As shown in **Figure 5A**, indicated by the green arrows, the original method tends to extract planar points as edge points, such as some ground points and certain quasi-planar points on banana leaves. In **Figure 5B**, our method demonstrates a more complete and uniformly distributed extraction of surface features, avoiding the adverse impact on mapping accuracy caused by the aggregation of numerous redundant feature points.

### 3.2 Point cloud preprocessing

**Figure 6:**
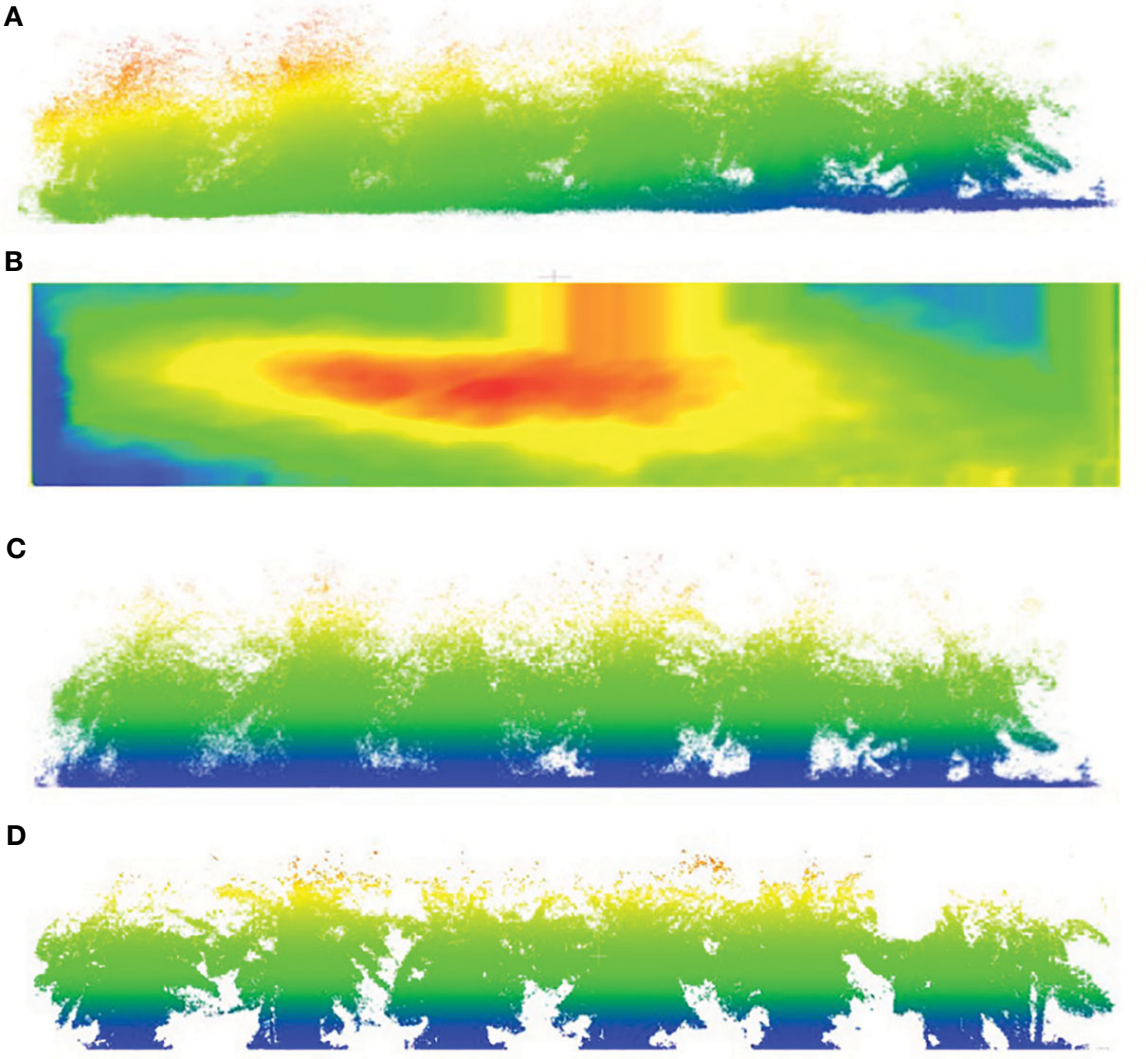
Point cloud preprocessing workflow. **(A)** Origin point cloud. **(B)** Ground surface fitting. **(C)** Normalize point cloud. **(D)** Filtered ground point cloud.

As shown in **Figure 6**, the reconstructed banana plantation contains a significant amount of noise and outliers, necessitating certain preprocessing steps for convenient subsequent usage. The preprocessing steps include the removal of point cloud noise, ground fitting, and ground correction. In this regard, we employed a widely used point cloud processing software (CloudCompare). **Figure 6A** illustrates the point cloud after denoising using statistical outlier removal (SOR), resulting in a reduction in the number of points from 1,931,651 to 1,755,892 without compromising quality. **Figure 6B** presents the ground surface model fitted using CSF. By adjusting various fitting parameters, we ensure that the extracted ground surface includes more ground points and fewer points corresponding to banana plants. To ensure consistent ground heights for different banana plants, we calculate the distance of each point to the surface and project it onto the plane. **Figure 6C** displays the banana plantation map after height correction. Although some ground points still exist in this image, we utilize the RANSAC plane filtering method to remove them, yielding a fully preprocessed point cloud as depicted in **Figure 6D**.

**Figure 7:**
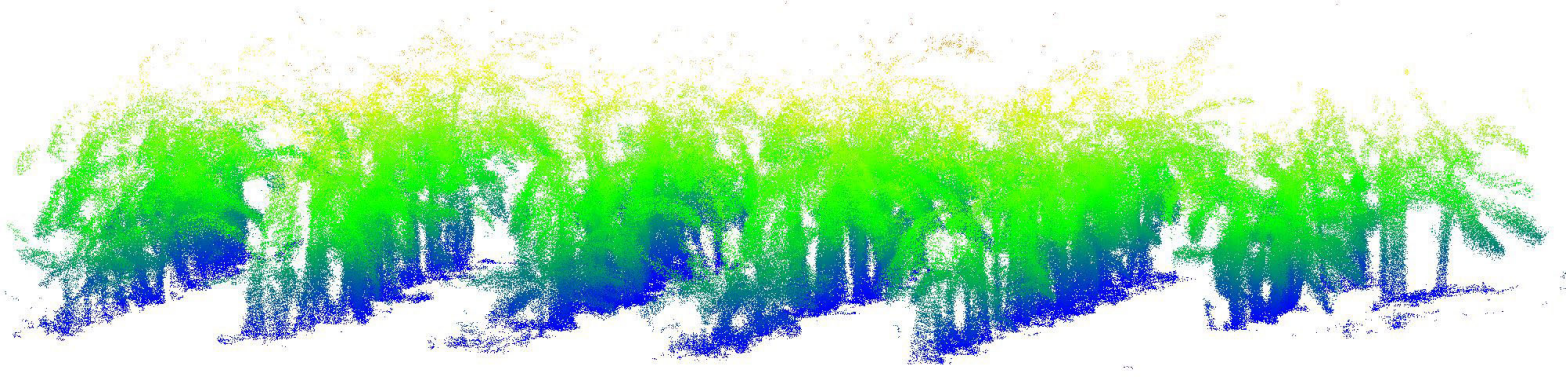
Banana plantation 3D point cloud map.

Using the distance-weighted feature extraction method proposed in Section 2.22, we constructed a banana plantation environment map. **Figure 7** illustrates the point cloud after preprocessing, revealing minimal alteration in the outer contours of the banana plants. The preprocessing, involving ground removal and point cloud normalization, effectively addressed the issue of inconsistent banana plant heights caused by terrain variations.

### 3.3 Banana plant point cloud segmentation

**Figure 8:**
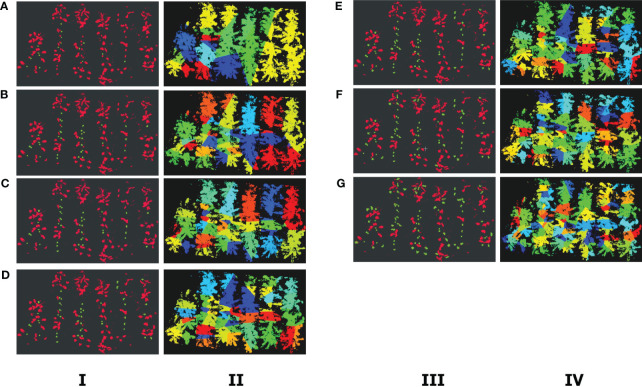
The results of Euclidean clustering and K-means clustering. In the Euclidean clustering plots in columns I and III, the green point clouds represent point clouds identified as banana pseudo-stems, whereas the red point clouds represent leaf points. In columns II and IV, the plots represent K-means clustering results under different T values. **(A)** T = 0.4 m. **(B)** T = 0.5 m. **(C)** T = 0.6 m. **(D)** T = 0.65 m. **(E)** T = 0.7 m. **(F)** T = 0.8 m. **(F)** T = 0.9 m. **(G)** T = 1.0 m.

The division of banana plants is also a crucial task in this article. To ensure the accuracy of banana plant segmentation, the combination of Euclidean clustering algorithm, threshold segmentation, and k-means clustering method is proposed in Section 2. 32, algorithm 1. We utilized Euclidean clustering for classifying the point cloud data at fixed heights, followed by joint threshold segmentation to extract pseudo-stem point clouds from the classification results and compute their pseudo-stem count and centroid coordinates. These obtained parameters were then employed in K-means clustering. The choice of K value significantly influences the effectiveness of K-means clustering. Therefore, we further investigated how to achieve the best clustering results under different threshold values, denoted as T. From **Algorithm 1**, the classification of banana pseudo-stems under different width thresholds (T) can be obtained, as shown in **Figure 8**. By experimentally varying the threshold segmentation parameter T after Euro-Clustering, the optimal parameters for segmenting adjacent plant point clouds in the banana plantation were obtained. Recent plant point clouds in the banana plantation were obtained.

Here, we used seven different T values to validate the results, and the experimental results are shown in **Table 1**. Through comparative experiments, it can be observed that when T = 0.6 m, the segmentation success rate is the highest. When T is greater than 0.6 m, it is unable to filter out the point clouds connecting adjacent banana plants, classifying the point cloud originally belonging to the banana pseudo-stem as leaf cloud, leading to a decrease in segmentation success rate. If the value of T is less than 0.6, the canopy point cloud will be segmented into multiple parts, classifying the leaf point cloud as pseudo-stem point cloud, resulting in a decrease in segmentation success rate. Therefore, this study selects T = 0.6 m as the experimental parameter.

**Table 1:** Segmentation success rate under different parameters.

Number	Parameter	Accuracy
1	T=0.4m	37.2%
2	T=0.5m	79.1%
3	T=0.6m	95.3%
4	T=0.65m	90.7%
5	T=0.7m	74.4%
6	T=0.8m	48.8%
7	T=1.0m	failed

### 3.4 Boundary point recognition method based on a continuous sliding window

**Figure 9:**
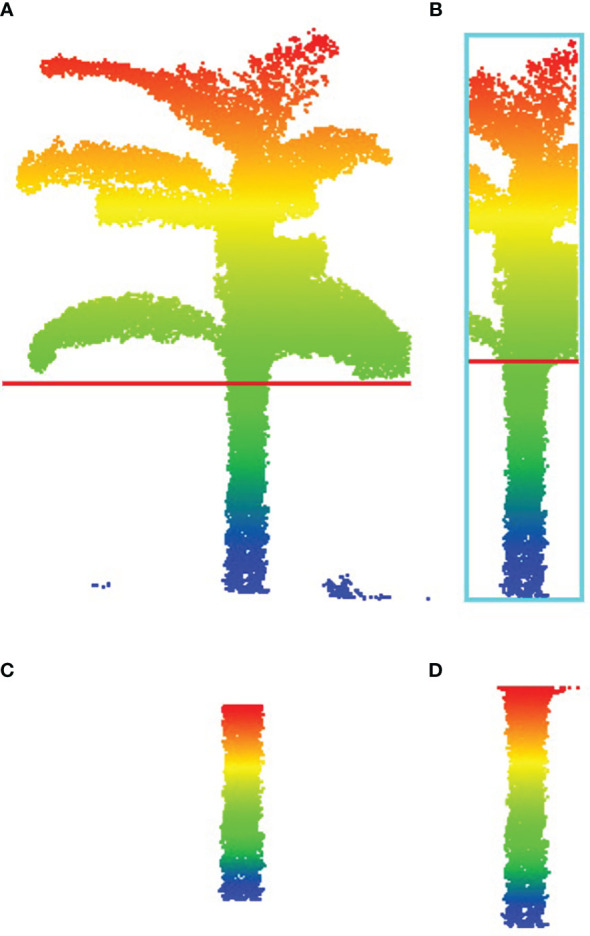
Distinguishing pseudo-stem point clouds using the continuity sliding window method. **(A)** Identification of stem-leaf junction points using the sliding window method. **(B)** Using the stem-leaf boundary points identified based on the continuous sliding window method. In both cases, the red line indicates the boundary point positions. **(C)** The pseudo-stem point clouds obtained by segmentation using the sliding window method. **(D)** Utilizing pseudo-stem point clouds segmented based on the continuity sliding window method.

Due to the interference caused by the drooping of the leaves, direct application of the horizontal slicing method in stem height measurement results in incomplete separation of the pseudo-stem, thereby affecting the accuracy of pseudo-stem height measurement. Therefore, it is necessary to eliminate redundant leaf clouds when measuring pseudo-stem height. This paper proposes a boundary point recognition method based on a continuous sliding window, as shown in **Algorithm 2**. **Figure 9C** depicts the pseudo-stem point cloud obtained by directly slicing and separating using the horizontal slicing method from **Figure 9A**. **Figure 9D** represents the complete pseudo-stem point cloud segmented by the method proposed in this paper, as shown in **Figure 9B**. By comparing **Figures 9C, D**, it can be observed that our method effectively reduces the occurrence of this situation, obtaining a more complete pseudo-stem point cloud.

### 3.5 Precision evaluation of diameter and height measurements of banana pseudo-stems

The experimental plot comprises a total of 43 banana plants. After European-style clustering classification, 39 banana plant diameter point clouds were identified, with four plants mistakenly classified as leaf point clouds. The main reason for the failure to identify certain banana plants is the loss of point clouds from pseudo-stems at a fixed height due to point cloud occlusion, resulting in smaller bounding boxes. Alternatively, the proximity of the pseudo-stem to a broken leaf can cause the bounding box to expand beyond its threshold range. To validate the accuracy of the computed pseudo-stem diameter and height parameters, a corresponding program was developed to automatically measure the banana pseudo-stem diameter. For the true measurement of banana diameter, we used a paper ruler to measure the circumference of the banana plant at three positions: 1.0 m, 1.1 m, and 1.2 m above the ground. We then calculated the average circumference and converted it into diameter. For the measurement of banana pseudo-stem height, we utilized a high-precision surveying ruler to repeat the measurement three times for each banana plant, from the ground to the point of first leaf insertion. The average of these measurements was taken as the true value of the pseudo-stem height. The accuracy of the measurement parameters was assessed using the root mean square error (RMSE), mean absolute percentage error (MAPE), and correlation coefficient (R) between the measured values and true values. The calculation methods are shown in Equations 21–23.


(21) $$RMSE=\sqrt{\frac{1}{n}\sum_{i=1}^{n}{\left(x_{mi}-x_{ai}\right)}^{2}}$$

(22) $$MAPE=\frac{1}{n}\sum_{i=1}^{n}\frac{\left|x_{mi}-x_{ai}\right|}{x_{mi}}\times 100\%$$

(23) $$R^{2}=\frac{Cov\left(x_{mi},x_{ai}\right)}{\sqrt{Var\left[x_{mi}\right]Var\left[x_{ai}\right]}}$$

#### 3.5.1 Measurement of pseudo-stem diameter parameters

**Figure 10:**
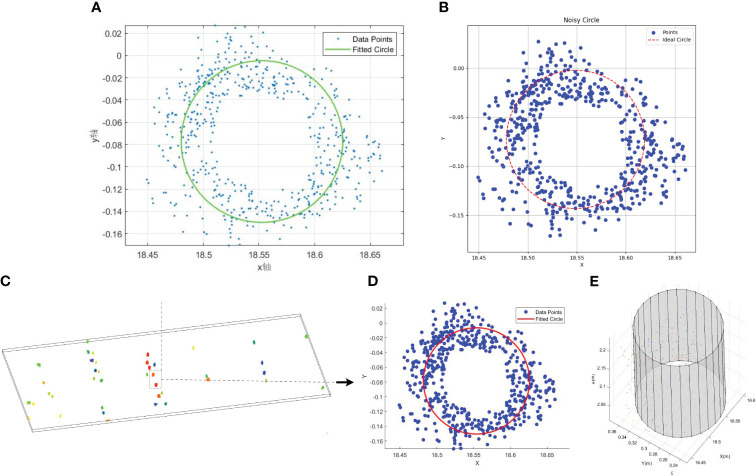
Different methods of circle fitting. **(A)** Least squares method. **(B)** Hough transform method. **(C)** Fixed-height point cloud. **(D)** Robust least trimmed squares method. **(E)** RANSAC 3D cylinder fitting method.

In the calculation of pseudo-stem diameter for banana plants, different methods exhibit varying degrees of robustness to noise and outliers. To obtain a more accurate estimation of banana pseudo-stem size, we compared four different circle fitting methods for calculating pseudo-stem diameter. As depicted in **Figure 10A**, we acquired point clouds of all banana pseudo-stems at a fixed height. In **Figure 10B**, the least squares circle fitting method was applied, whereas in **Figure 10C**, the Hough Transform circle fitting method was employed. In **Figure 10D**, the robust least trimmed squares method was utilized, and in **Figure 10E**, the RANSAC cylindrical fitting method was used to estimate the sizes of all banana pseudo-stems’ diameters.

This comparison aims to assess the influence of these four fitting methods on the accuracy of pseudo-stem diameter determination, considering their robustness to noise and outliers. The results presented in the figures allow for a visual examination of the differences in the estimated pseudo-stem sizes between the four fitting methods.

**Figure 11:**
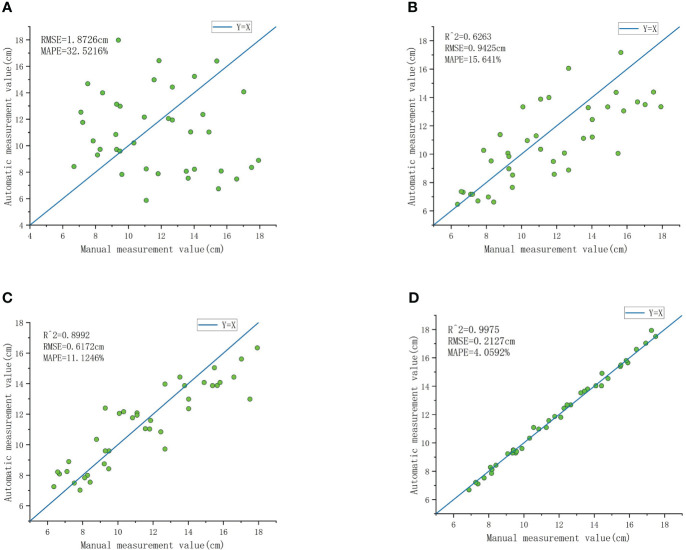
Comparison of measurement results between RANSAC cylinder fitting and least squares circle fitting. **(A)** The result of measuring pseudo-stem diameter based on the least squares circle fitting. **(B)** The measurement results obtained by Hough transform circle fitting method. **(C)** Comparison between the measured value and the real value of the robust least trimmed squares circle fitting method. **(D)** Results obtained from measuring pseudo-stem diameter based on RANSAC cylinder fitting.

**Figure 11A** shows the pseudo-stem diameters computed using the least squares fitting method, where *R* ^2^ is a negative value and deemed meaningless, hence not annotated in the figure. The RMSE is 0.018726 cm, and the MAPE exceeds 32%. **Figure 11B** depicts the pseudo-stem diameters measured using the Hough Transform circle fitting method, with an *R* ^2^ of 0.6263, RMSE of 0.9425 cm, and MAPE of 15.641%. **Figure 11C** shows the measurement results obtained using the robust least trimmed squares method, with an *R* ^2^ of 0.8992, RMSE of 0.6172 cm, and MAPE of 11.1246%. **Figure 11D** illustrates the pseudo-stem diameters obtained using the RANSAC fitting method, where *R* ^2^ *>* 0.99, RMSE is controlled within 0.0022 m, and MAPE is less than 4.1%. The automatic measurement accuracy of banana pseudo-stem diameter exceeds 95.9%. Clearly, the pseudo-stem diameter measurements obtained through the least squares method exhibit significant errors and lack practical significance, whereas the errors in the measurements obtained through the Hough Transform and robust least trimmed squares methods are greater than those obtained through the RANSAC circle fitting method. This comparative experiment demonstrates that, for the measurement of banana pseudo-stem size, the RANSAC cylindrical fitting method possesses high robustness, mitigating the impact of surface outliers on measurement results. Employing this method results in precise measurements of banana pseudo-stem diameter, with algorithmic automated values closely aligning with the ground truth obtained from manual point cloud measurements.

#### 3.5.2 Measurement of pseudo-stem height parameters

**Figure 12:**
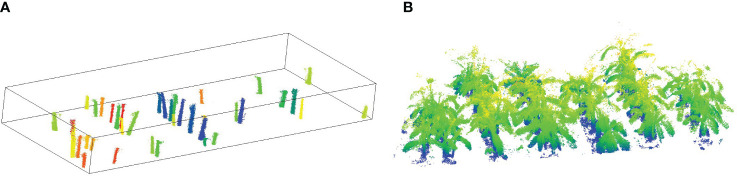
**(A)** Extracted pseudo-stem point cloud using the continuous sliding window method. **(B)** Corresponding 3D point cloud map.

After fitting three-dimensional cylinders to the point cloud of fixed-height pseudo-stems, separating banana plants using K-means clustering, and applying threshold segmentation to remove most of the non-pseudo stem points, we employed the continuity-based sliding window method proposed in this paper to identify the boundary points between banana leaves and pseudo-stems. The complete pseudo-stem point cloud was extracted, and the results are shown in **Figure 12**. Using the extracted point cloud, we measured the height of banana pseudo-stems. We also compared the heights measured using the sliding window method with the results obtained from the continuity-based sliding window method proposed in Section 2.42 of this paper. In the experimental field, we measured the pseudo-stem height of 43 banana plants, with only three plants being misidentified. The pseudo-stems of 39 banana plants were correctly identified, and the measurement results were within the threshold.

**Figure 13:**
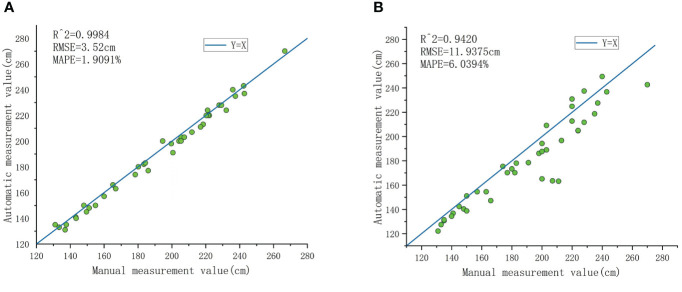
Comparing the results of using a continuous sliding window and a regular sliding window. **(A)** Presenting the results of pseudo-stem height measurements obtained through the continuous sliding window method. **(B)** The results of pseudo-stem height measurements using the sliding window method.

As shown in **Figure 12**, the program developed for the automatic and accurate measurement of banana pseudo-stem height in the experimental field was utilized, and the results were compared with manually measured true values. **Figure 13A** displays the results of banana pseudo-stem height measured using the continuity-based sliding window method, with an *R* ^2^ greater than 0.99, RMSE controlled within 0.036 m, and MAPE not exceeding 2%. **Figure 13B** shows the results of banana pseudo-stem height measured using the sliding window method, with an *R* ^2^ of 0.94, RMSE of 11.94 cm, and MAPE of 6.04%. Consequently, the proposed method for measuring banana pseudo-stem height demonstrated high accuracy, with algorithmic measurements closely aligning with manual measurements.

### 3.6 Discussion

This study investigates the Synchronous Localization and Mapping (SLAM) method based on handheld mobile laser for point cloud mapping, successfully applied to the construction of a point cloud map for a banana plantation. Considering the complex environment of the banana plantation, with features like leaf occlusion, we propose a combined method using filtering, Euclidean clustering, and K-Means clustering for segmentation of banana plants. This method successfully segments banana plants and calculates the diameter of the pseudo-stem. An approach based on continuity sliding window is introduced to enhance the accuracy of identifying boundary points between the pseudo-stem and leaves, along with the computation of pseudo-stem height. Experimental results demonstrate the effectiveness of the proposed method in measuring phenotypic parameters on a large scale in banana plantations, providing valuable references for orchard management.

Plant point cloud segmentation: To separate banana plants from the point cloud map, we propose a threshold segmentation method that combines the Euclidean clustering and K-Means clustering techniques. However, K-Means is influenced by the number of clusters (K) and the initial cluster centers (C). Therefore, we explore the impact of different threshold parameters on the number of clusters (K) and determine the optimal threshold parameter (T). As shown in **Figure 8**, we obtain different results for Euclidean clustering and K-Means clustering by varying the value of T. When the threshold T is too small, the Euclidean clustering may classify some pseudo-stem point clouds as leaf point clouds, and when T is too large, leaf point clouds may be classified as pseudo-stem point clouds, affecting the accuracy of clustering. By comparing the results, we determine the optimal threshold T, providing accurate initial results for subsequent phenotype parameter measurements.

Boundary point recognition based on continuous sliding window: Accurately identifying the boundary between the pseudo-stem and leaves is a crucial aspect for providing precise measurements of pseudo-stem height. The continuity-based method proposed in this paper addresses recognition errors caused by the non-smooth surface of the pseudo-stem or protrusions. As shown in **Figure 9C**, the traditional sliding window method, when encountering abrupt changes in perimeter, directly identifies it as a boundary point, resulting in incomplete recognition of pseudo-stem points and affecting the measurement accuracy of pseudo-stem height. However, the method proposed in this paper effectively resolves this issue, as depicted in **Figure 9D**, showcasing the successfully and comprehensively identified pseudo-stem point cloud. This has a significant impact on the subsequent calculation of pseudo-stem height.

Accuracy evaluation of pseudo-stem diameter measurement: To evaluate the robustness of the proposed method, we compared the results of pseudo-stem diameter calculation based on the least squares method, Hough transform circle fitting method, robust least trimmed squares circle fitting method, and RANSAC cylinder fitting method. As shown in **Figure 11**, the pseudo-stem diameters calculated using the RANSAC circle fitting method are closer to manual measurements compared to the other three methods, with the least squares method yielding more dispersed results that deviate significantly from the true values. From a technical perspective, there are several reasons for this. Firstly, the typical measurement accuracy of lidar is approximately 2 cm, leading to maps that may contain a large number of discrete points. Secondly, banana plants often exhibit certain degrees of inclination, resulting in a considerable number of discrete points when projecting the point cloud from a fixed height onto the XY plane. The least squares method aims to find the circle that minimizes the sum of squared distances between the circle and data points through mathematical optimization. Therefore, it may perform poorly on datasets containing numerous discrete points. Conversely, the robust least trimmed squares method achieves more accurate measurements by trimming points with large residuals and iteratively fitting the remaining points. The Hough Transform circle fitting method is similarly insensitive to discrete points, but it requires prior information such as the initial coordinates of the circle center and the initial diameter, and the accuracy of this prior information significantly influences its measurement results. On the other hand, the RANSAC fitting algorithm estimates model parameters by randomly selecting data points and evaluates model quality based on the error between data points and the model. Therefore, it exhibits higher robustness and measurement accuracy when dealing with datasets containing noise and outliers. In comparison with Kinect v2 measurements (**Table 2**, number 1 and 2), the MAPE of our method increased by 1.7% to 2.8%, which is within a reasonable range. While camera-based measurements slightly improve the accuracy of pseudo-stem diameter, using a camera introduces challenges such as the need to control sampling distance for optimal measurement, susceptibility to environmental changes leading to significant measurement errors, and difficulties in implementing large-scale measurements. Comparing with ground-based LiDAR measurements (**Table 2** number 3 and 4), the measurement method proposed in this paper (**Table 2**, number 5) shows an increase in MAPE of 2.76% to 3.02%, which is attributed to the fact that the measurement error of the lidar device itself is approximately 10 times that of the camera and ground-based lidar.

**Table 2:** Comparison of the accuracy of the proposed method with other studies.

Parameters	Number	RMSE	MAPE
Pseudo-stem diameter	1 2 3	2.70 mm 4.06 mm 3.80 mm	1.40% 1.40% 1.30%
	4	3.90 mm	1.04%
	5	2.13 mm	4.06%
Pseudo-stem height	6 7	4.50 cm 20.14 cm	6.32% 5.11%
	8	27.90 cm	9.40%
	9	3.52 cm	1.91%

Number 1 and 6 contributed by Wang et al. (2022), numbers 2 contributed by Song (2019), numbers 3, 4, 7, and 8 contributed by Miao (2022), and numbers 5 and 9 contributed by this study.

Accuracy evaluation of pseudo-stem height measurement: To validate the impact of the continuity-based sliding window method on the measurement of pseudo-stem height, we compared the results with the conventional sliding window approach, as shown in **Figure 13A**. The measurements based on the proposed method in this study are relatively close to the ground truth, indicating accurate recognition of boundary points. In contrast, the measurements from the conventional sliding window method are generally lower than the actual values, primarily due to the unevenness of the pseudo-stem. Instances of protrusions are often incorrectly identified as boundary points, leading to consistently lower measurements and demonstrating the poor robustness of this method. The average measurement error for banana plant height is 4.5 cm, with an average measurement error of 6.32% (**Table 2**, number 6). In this study, the average relative error for height measurement is 1.91%, representing an improvement of approximately 4.4% in measurement accuracy compared to camera-based measurements. This improvement is attributed to the increasing distance between Kinect and the measured plant, causing a proportional increase in measurement errors and a decrease in measurement correlation. However, the variation in measurement errors is relatively small when the distance change is not significant. In comparison with ground-based LiDAR measurements (**Table 2**, numbers 7 and 8) for two experimental sites, the results for pseudo-stem height measurement are as follows: 1) RMSE of 0.2014 m, MAPE of 5.11% for the first site, and 2) RMSE of 0.2788 m, MAPE of 9.40% for the second site. Compared to the measurement method presented in this study (**Table 2**, number 9), our approach shows a reduction in MAPE for stem height measurement ranging from 3.2% to 7.5% across the two experimental sites, and both sets of measurements fall within a reasonable range.

The comparison results indicate that the methodology proposed in this paper slightly falls short in measuring the diameter of banana pseudostems when compared to camera and ground-based lidar methods. However, it surpasses these methods in the accuracy of measuring pseudo-stem height. Part of the reason for this discrepancy lies in the differing errors associated with the measurement equipment. Unlike expensive ground-based lidar devices that can achieve a measurement accuracy of 1 mm–2 mm, and cameras whose measurement error typically ranges from 2 mm–4 mm (with errors increasing as the measurement distance grows), the mobile lidar used in this study has a measurement accuracy of ±2 cm. Given that the diameters of banana plants collected in the field generally fall between 10 cm–18 cm, the precision of the equipment significantly impacts the measurement of smaller targets like banana diameters. For high-precision devices, measuring the pseudo-stem diameter using the least squares method is feasible, but this is not suitable for the pseudo-stem diameter measurements discussed in this paper. Therefore, we employ a three-dimensional cylindrical fitting method to minimize the impact of equipment precision on the accuracy of diameter measurements. The accuracy of measuring pseudo-stem height is less affected by equipment precision, and the integrity of pseudo-stem point cloud segmentation can enhance measurement accuracy. The segmentation method based on a continuity sliding window proposed in this paper can extract a complete pseudo-stem point cloud, thereby providing superior accuracy in pseudo-stem height measurements compared to camera and ground-based lidar methods.

Time consumption: Compared to manual measurement, the proposed method requires 8 min for a single scan, with 7 min for point cloud preprocessing and program execution, totaling 15 min. Manual measurement for each banana plant takes approximately 3 min, resulting in a total of 123 min for 41 plants. The proposed method is approximately 1/8th of the time required for manual measurement, effectively improving orchard management efficiency.

## 4 Conclusions

The text describes the development of a handheld mobile scanning system combining mobile LiDAR and IMU sensors for the purpose of mapping banana plantations. Additionally, an automated algorithm for measuring the diameter and height of banana pseudo-stems is designed. The results indicate an absolute average error of 0.2127 cm and a relative average error of 4.06% for banana pseudo-stem diameter measurement, with a correlation coefficient of 0.99. The average absolute error for banana pseudo-stem height measurement is 3.52 cm, with an average relative error of 1.90% and a correlation coefficient of 0.99. The overall error meets surveying requirements. Research has shown that the method proposed in this paper is applicable for extracting tree phenotypic parameters in complex and obstructed orchard environments, thereby offering orchard managers a novel approach for phenotypic measurement. In the future, the integration of GPS data will be implemented to achieve precise localization for each banana plant.

## Data availability statement

The raw data supporting the conclusions of this article will be made available by the authors, without undue reservation.

## Author contributions

ZY: Project administration, Writing – review & editing. QJ: Conceptualization, Methodology, Software, Validation, Writing – original draft. JD: Data curation, Investigation, Visualization, Writing – review & editing. MJ: Data curation, Investigation, Visualization, Writing – review & editing. HF: Data curation, Investigation, Visualization, Writing – review & editing. XX: Formal analysis, Software, Writing – review & editing.
